# Country-Level Factors Associated With Nurse Salaries: Empirical Evidence From Organisation for Economic Co-operation and Development Countries and Taiwan

**DOI:** 10.1097/jnr.0000000000000585

**Published:** 2023-11-17

**Authors:** Yu-Hung CHANG, Chia Hui HSU, Yu-Chun TSENG, Chao A. HSIUNG

**Affiliations:** 1 PhD, Project Assistant Investigator, Institute of Population Health Sciences, National Health Research Institutes, Zhunan, Miaoli County, Taiwan; 2 MS, Research Assistant, Institute of Population Health Sciences, National Health Research Institutes, Zhunan, Miaoli County, Taiwan; 3 PhD, Honorary Investigator, Institute of Population Health Sciences, National Health Research Institutes, Zhunan, Miaoli County, Taiwan.

**Keywords:** nurse salary, nursing shortage, nursing workforce, health policy

## Abstract

**Background:**

Salary impacts nurse retention rates and thus is a factor affecting the nursing shortage both in Taiwan and around the world. Nurses in Taiwan earn a low salary compared with other health professionals and may be undervalued compared with their international counterparts.

**Purpose:**

This study was designed to analyze the factors associated with nurse salary (NS) in Organisation for Economic Co-operation and Development (OECD) countries and to compare NS in Taiwan with those in OECD member states.

**Methods:**

Data were extracted from the OECD statistics database and official statistics for Taiwan. For the 28 OECD member countries considered in this study and Taiwan, 21 indicators characterizing healthcare systems, including demographics, socioeconomic status, health behaviors and risks, healthcare resources, health financing, healthcare utilization, health outcomes, and economic inequality, were examined for the period of 2009–2018. A random-effects model (REM) and a fixed-effects model (FEM) were used to investigate the associations between these indicators and annual NS levels. The expected annual NS for Taiwan was estimated and compared with the actual NS for Taiwan using the REM.

**Results:**

In the REM, higher NS in OECD countries was shown to be positively associated with gross domestic product per capita (0.49, 95% confidence interval [CI] [0.41, 0.56]), proportion of population aged 65 years and over (2.72, 95% CI [2.17, 3.26]), crude birth rate (1.02, 95% CI [0.56, 1.49]), number of computerized tomography scanners per million population (0.26, 95% CI [0.17, 0.35]), alcohol consumption per person (0.94, 95% CI [0.26, 1.61]), and prevalence of obesity (0.64, 95% CI [0.40, 0.89]) and to be in inversely associated with infant mortality rate (−3.13, 95% CI [−3.94, −2.32]), bed density (−0.99, 95% CI [−1.72, −0.25]), number of hospital discharges (−0.08, 95% CI [−0.11, −0.05]), household out-of-pocket expenditure as a percentage of health expenditure (−0.34, 95% CI [−0.56, −0.11]), and the Gini coefficient (−0.25, 95% CI [−0.50, −0.01]). The FEM results were similar to those of the REM. The predicted annual NS for Taiwan based on the REM rose from 29,390 U.S. dollars (corrected for purchasing power parity; 95% CI [22,532, 36,247]) in 2009 to 49,891 U.S. dollars (95% CI [42,344, 57,438]) in 2018. The actual annual NS in Taiwan in 2018 was approximately 12% lower than the model-predicted value.

**Conclusions/Implications for Practice:**

Taiwan has a lower NS compared with its OECD counterparts. These findings may help policymakers, healthcare managers, and nurse organizations develop effective strategies to improve the remuneration system for nurses in Taiwan.

## Introduction

The current global shortage of health professionals is particularly severe with regard to nurses. According to World Health Organization (WHO, 2020) estimates, the global shortage of nurses was approximately 5.9 million in 2018 and was expected to be 5.7 million in 2030. A study of 23 member states of the Organisation for Economic Co-operation and Development (OECD) projected that these countries would have a shortage of 2.5 million nurses by 2030 (Scheffler & Arnold, 2019). Bridging this shortfall is both increasingly important because of global population growth and rapid population aging and critical to achieving health-related Sustainable Development Goals (WHO, 2020).

Nursing staff shortages are associated with adverse patient outcomes, with lower nursing staffing in hospitals contributing to higher hospital mortality (Aiken et al., 2018; Griffiths et al., 2019). A recent systematic review that evaluated 18 studies on the association between nurse staffing and missed care found that 14 of the 18 reported an association between lower nurse staffing and more reports of missed care (Griffiths et al., 2018). Furthermore, an umbrella review summarized the findings of 15 previous literature review studies and concluded there is strong evidence of an association between nurse staffing levels and length of stay, patient satisfaction, quality of nurse-delivered care, and likelihood of readmission (Blume et al., 2021). Meanwhile, a cross-country study found that higher nurse densities are associated with higher life expectancy (Amiri & Solankallio-Vahteri, 2019). Similarly, an analysis of data for 35 OECD countries reported that, in the long term, a 1% increase in nurse density is associated with 0.98%, 0.97%, and 0.96% reductions, respectively, in infant mortality, neonatal mortality, and perinatal mortality (Amiri et al., 2020). Finally, in addition to health outcomes for patients and populations, inadequate nurse staffing has been linked to negative outcomes for nurses. Lower rates of nursing staffing increase the risks of burnout, job dissatisfaction, and intention to leave (Dall'Ora et al., 2020; Shin et al., 2018).

Adequate salary significantly impacts nurses' motivation, performance, productivity, job satisfaction, and retention (McHugh & Ma, 2014; Muthmainnah & Hariyati, 2018). For health professionals, low pay reduces job satisfaction (Halcomb et al., 2018), and low rates of satisfaction negatively impact retention (Bimpong et al., 2020). In contrast, higher salaries and benefits for nurse practitioners have been associated with longer employment durations (Hagan & Curtis, 2018). However, raising salaries alone may have a limited effect on retention (Bimpong et al., 2020).

For nurses who are new to the profession, dissatisfaction with salary is the main factor cited in those considering a career change. Young nurses weigh their salary against the demands and responsibilities of their work (Flinkman et al., 2008). Attrition among young nurses reduces the number of nurses available to replace nurses who retire and those who leave the workforce before retirement age (Flinkman et al., 2008). In addition, the existence of salary compression (i.e., the lack of significant salary differentiation throughout one's career) in nursing can make the career unattractive to recruits and, among experienced nurses, reinforce dissatisfaction and intention to leave (Greipp, 2003).

International migration of the nursing workforce is another issue exacerbating the nursing shortage in certain regions, as nurses in lower-wage countries are enticed to seek nursing employment in higher-wage countries. Differences in nursing wage levels between countries are one of the main drivers of such migration (Awases et al., 2004; Dovlo, 2007). Nurses in developing countries leave their home countries to search for better professional development, salaries, working conditions, and living standards in developed countries that are experiencing nursing workforce shortages (Kline, 2003). The migration of health professionals particularly impacts developing countries with more fragile healthcare systems. For those who remain in their home countries, international emigration increases the workload and risk of overwork, making retention of healthcare professionals more challenging (Tomblin Murphy et al., 2016).

Over the past decade in Taiwan, it has been noted that the salary of nurses is low compared with those of physicians and other medical and nonmedical professionals (Fan & Chen, 2009). Moreover, amidst the 2019 coronavirus pandemic, high job stress and stagnant growth in nurses' wages were acknowledged by national legislators as essential factors contributing to nursing turnover (Huang, 2021). To determine whether and to what extent nurses in Taiwan are undervalued in terms of their salaries, cross-country comparisons may provide a useful reference. Few studies have adopted a cross-country approach to examine the determinants of nurse salary (NS) across different healthcare systems. Moreover, even fewer have examined this issue quantitatively.

This study was developed to examine country-level indicators associated with NS using OECD country data and to compare NS in Taiwan with those in OECD countries. An explanatory model for NS was developed based on OECD data and indicators commonly used in the literature to compare healthcare systems. The predictions of the model were then compared with actual NS rates in Taiwan.

## Methods

A variable-oriented approach was used to identify health system indicators significantly associated with NS and to compare the NS level in Taiwan with those of counterpart OECD countries. The variable-oriented approach is mainly used to assess relationships between case (e.g., country) variables in large, “observational” samples with the goal usually being to specify general patterns that apply to the sample as a whole to make inferences and predictions (Ragin, 1999). The application of the variable-oriented approach to cross-country comparisons of health systems may be attributed to research in health economics (Cacace et al., 2013). This approach involves selecting data such as databases, comparators, and variables and using a quantitative method to identify links between variables. In the following subsections, we introduce the selection of data and variables and the statistical analysis methods used to build NS prediction models feasible for comparing NS levels between OECD countries and Taiwan.

### Data

Data were extracted for this study from the OECD.Stat database (https://stats.oecd.org/) for the period of 2009–2018 for the following 28 OECD countries: Australia, Belgium, Canada, Czech Republic, Denmark, Estonia, Finland, France, Germany, Greece, Hungary, Iceland, Ireland, Israel, Italy, Japan, Korea, Mexico, Netherlands, New Zealand, Norway, Poland, Slovak Republic, Slovenia, Spain, Turkey, the United Kingdom, and the United States. Ten of the 38 OECD member countries were not included in this study because of missing data on crucial study variables. Most of the 28 countries selected were high-income countries and considered to be at an equivalent or higher level of development than Taiwan.

We collected indicators from the OECD.Stat database covering the themes of health, demography and population, national accounts, and social protection and well-being. These four themes contain most of the indicators related to the study topic. Although data collection methods and definitions used vary across these countries, the database provides high-quality data for comparing healthcare systems between countries (Reibling et al., 2019). The relevant statistics and indicators for Taiwan were obtained from the official websites of the Directorate General of Budget, Accounting and Statistics of the Executive Yuan, the Ministry of Health and Welfare, and the Ministry of Labor, Taiwan.

OECD.Stat was used as the primary data source to ensure consistency in terms of variable definitions, data collection methods, and data quality. There were missing values for some indicators in the OECD.Stat database. To obtain the data for these values, we consulted the Health at a Glance report, which is published every 2 years by the OECD (https://www.oecd.org/health/health-at-a-glance/). Additional missing data for the OECD countries in Europe were also obtained from Eurostat (https://ec.europa.eu/eurostat/web/health/overview) and from the official WHO website (https://www.who.int/data/gho) or official government statistics for non-European OECD countries. OECD.Stat and Eurostat cover a wide range of themes and indicators at the country level. We have compared them based on data of the common member countries and found consistency between them. The WHO database focuses on health-related themes. In this study, only a few missing values were replaced with the WHO data. After consulting the abovementioned sources, values for data still unavailable were imputed using the linear interpolation method and multivariate imputation using chained equations (Van Buuren, 2018).

The data in the abovementioned, publicly available databases were aggregated and cannot be used to identify individuals. Analyses based on these data do not involve human subject research.

### Dependent Variable

The average annual NS for the OECD countries was treated as the dependent variable (DV) in this study. This salary was calculated as the average total annual income, including social insurance and income taxes. The calculation also included nonrecurring payments such as bonuses and nightshift and overtime compensation. The data included nurses who were public or private employees. However, in countries with predominantly publicly funded healthcare systems such as the United Kingdom, New Zealand, and Denmark, the data included nurses working in public healthcare institutions only. For most countries, the data only covered nurses working full-time in hospitals, although, for some countries and for certain years, the data also included nurses working in other settings and part-time nurses. Furthermore, for some countries, the data included only registered nurses, whereas others also included data on lower-level nurses. We did not further adjust the data for the above differences because of a lack of breakdown information. The average NS for each country was converted into U.S. dollars (USD) and adjusted for purchasing power parity (PPP).

The average NS level in Taiwan was calculated by consulting the Ministry of Labor's “Survey on Earnings by Occupation” (https://pswst.mol.gov.tw/psdn/). This survey is conducted each year in August. Thus, July was used as the reference period for each year's data. We extracted the data for nurses working in the healthcare system. Nurses' earnings comprised regular and nonregular earnings. We multiplied the monthly earnings by 12 to estimate annual salary and added 1.5 months of regular earnings to represent a year-end bonus. The annual NS level for Taiwan was also converted into USD and adjusted for PPP.

### Independent Variables

To identify the indicators associated with NS, published research on comparative health systems and the health workforce was consulted. Salary level is generally determined by the supply of and demand for workers in the market, and the OECD proposed a general framework for assessing the main factors affecting the supply and demand of health workers (Ono et al., 2013). According to this framework, demand for health workers is derived from the demand for healthcare, affected by demographics, morbidity and associated health risks, the purchasing power for healthcare (gross domestic product [GDP] and health financing), and healthcare utilization, whereas supply of health workers is determined by the stock, inflow, and outflow of human resources for health (Ono et al., 2013), which are impacted by economic inequality (Squires et al., 2016). As the health system transforms inputs such as human resources, capital, and technology into outputs (i.e., population health; Frogner et al., 2015), the scope of the variable selection was extended in this study to encompass population health outcomes. Considering the relevance to NS based on the literature and theoretical grounds and the availability and completeness of data across countries, we selected 22 indicators from the OECD.Stat database and grouped them into seven dimensions, including demographics, socioeconomic status, health behaviors and risks, healthcare resources/input, health financing, healthcare utilization, population health outcomes, and economic inequality.

Demographics collected included the proportion of the population aged 65 years and over and the crude birth rate. Meanwhile, the socioeconomic context was represented by GDP per capita and the proportion of the population aged 15–64 years with a tertiary education. Health behaviors and risks comprised the prevalence of smoking, per capita alcohol consumption, and the prevalence of obesity.

Healthcare resources/inputs were categorized into health professional human resources, capital inputs, and the use of medical technology. Physician density and nurse density represented health professional human resources. Hospital bed density represented the capital inputs of a healthcare system. Computerized tomography scanners per million population represented the use of medical technology. Health financing was measured using health expenditure as a percentage of GDP and household out-of-pocket expenditures as a percentage of total health expenditures. The former represented the overall size of the healthcare market, whereas the latter represented the public/private-sector balance of the healthcare system.

This study took the number of doctor consultations per capita, the number of hospital discharges, and the average length of stay in hospital as indicators of health service utilization. The three indicators used to represent population health outcomes included the crude death rate, infant mortality rate, and life expectancy at birth. In addition, the two indicators used to represent healthcare quality were the 30-day in-hospital mortality rate for acute myocardial infarction (AMI) after hospital admission (indicating acute care quality in hospitals) and the hypertension hospital admission rate (representing the rate of avoidable hospital admissions and indicating primary care quality).

Economic inequality was indicated by the Gini coefficient and the gender wage gap. The Gini coefficient, ranging between 1 and 0, measures the degree to which incomes are concentrated or equally distributed across members of society, with values closer to 1 indicating incomes are more unequally distributed. The gender wage gap was defined as the difference between the median incomes of men and women relative to the median income of men.

The definitions and dimensions of the variables are summarized in Table 1. The means and standard deviations of these variables for the OECD countries and Taiwan are reported.

**Table 1.** Description and Summary Statistics of Study Variables Across the Selected OECD Countries and Years, 2009–2018

Dimension and Variable Name	Variable Description, Definition, and Relevance to Demand/Supply of Healthcare and Nurses	OECD Countries (*N* = 28; *n* = 280)		Taiwan (*N* = 1; *n* = 10)	
		Mean	*SD*	Mean	*SD*
Dependent variable					
NS	Annual nurse salary, 1,000 USD, PPP	42.22	14.86	38.06	3.19
Demographic					
AGE65	Proportion of the population aged 65+ years. An indicator of population aging indicating an increased demand for healthcare.	9.08	2.35	12.11	1.40
CRBIRTHR	Crude birth rate, annual number of live births per 1,000 population. Infants and children have higher healthcare needs compared with adults.	11.72	3.18	8.53	0.75
Socioeconomic context					
GDP	GDP per capita, 1,000 USD, PPP. It indicates the purchasing power for services provided by health professionals.	38.57	11.76	43.78	5.05
HIEDU	Proportion of the population aged 15–64 years with tertiary education. Higher education helps to produce health professionals.	33.97	10.47	40.60	3.32
Health behaviors and risks					
SMOKER	Proportion of population aged 15+ years who are daily smokers. A higher prevalence rate results in increased demand for healthcare.	19.26	5.02	16.93	2.32
ALCHCONS	Liters of pure alcohol per capita of aged 15+ years. A greater consumption results in increased demand for healthcare.	8.74	2.57	3.19	0.47
OBESEP	Self-reported, proportion of population aged 15+ years with a body mass index of 30 or over. A higher prevalence rate results in increased demand for healthcare.	16.68	5.58	15.59	1.22
Healthcare resources/input					
NURSENO	Nurse density, the number of practicing nurses providing services directly to patients, per 1,000 population. Increased nurse workforce supply may reduce nurses' salaries.	8.75	3.96	6.15	0.54
DOCTORNO	Physician density, the number of practicing doctors providing services for individual patients, per 1,000 population. It possibly increases demand for nursing staff when there is a shortage of physicians.	3.21	0.86	1.82	0.12
HBED	Hospital beds regularly maintained and staffed and immediately available for the care of admitted patients, per 1,000 population. It may increase the need for nursing staff according to medical regulations.	4.78	2.66	4.21	0.06
CTS	Computed tomography scanners per million population. The use of medical technology represents the efficiency of healthcare.	24.56	19.90	15.43	1.15
Health financing					
EXPHEAL	Health expenditure as a percentage of GDP. The purchasing power for healthcare.	8.87	2.30	6.38	0.16
OOPP	Household out-of-pocket expenditure as a percentage of health expenditure. Increased out-of-pocket may decrease the demand for healthcare.	18.97	7.62	34.28	0.76
Healthcare utilization					
LOS	Average length of stay, calculated by dividing the number of bed days by the number of discharges during the year. Intermediate output of nurses' labor inputs; it may reflect quality of care.	6.44	2.37	8.94	0.23
CONSULTD	Number of physician consultations per capita. Intermediate output of nurses' labor inputs.	6.94	2.99	12.47	0.42
HDISCHAR	Inpatient discharge, release of a patient who was formally admitted into a hospital for treatment and/or care and who stayed for a minimum of one night, per 1,000 people. Intermediate output of nurses' labor inputs.	151.67	41.40	134.43	3.88
Health outcomes					
CRDTHR	Crude death rate, annual number of deaths per 1,000 population. Final output of nurses' labor inputs.	8.61	2.05	6.83	0.41
INFMORTR	Infant mortality, per 1,000 live births. Final output of nurses' labor inputs.	2.74	1.65	2.45	0.16
LE	Life expectancy at birth. Final output of nurses' labor inputs.	80.17	2.49	79.80	0.57
AMI	In-hospital mortality rates within 30 days after admission for acute myocardial infarction (AMI), per 100 patients. The quality indicator for acute care of hospitals.	7.42 ^a^	4.55	8.00 ^c^	
ADMRHYPT	Hypertension hospital admission, annual number of nonmaternal/nonneonatal hospital admissions with a principal diagnosis code of hypertension, per 100,000 population. The quality indicator for primary care.	83.19 ^b^	84.50	9.40 ^d^	
Economic inequality					
GINI_PC	The Gini coefficient. Economic inequalities can increase financial barriers to healthcare access and reduce demand for healthcare.	31.18	5.15	33.88	0.31
GWAGEGAP	Gender wage gap, difference between the median income of men and women relative to the median income of men, 1,000 USD, PPP. Nurses are more likely to be female, and the lower gender wage gap means nurses are better paid.	13.76	7.34	15.88	1.23

*Note. N* = number of countries; *n* = number of observations; OECD = Organisation for Economic Co-operation and Development; USD = U.S. dollars; PPP = purchasing power parity; GDP = gross domestic product.

^a^Because of missing data for some countries and periods, the number of countries is *N* = 27 and the number of observations is *n* = 243. ^b^Because of missing data for some countries and periods, the number of countries is *N* = 26 and the number of observations is *n* = 229. ^c^Data for year 2013 only. ^d^Data for year 2015 only.

### Statistical Analysis

In this study, both the DV and independent variables (IVs) were repeatedly measured across multiple countries, and panel regression was used for the analysis. In this study, the random-effects model (REM) was used to consider both intercountry and intracountry (between and within) variations, whereas the fixed-effects model (FEM) was used to explore intracountry associations between the DV and IVs, controlling for fixed intercountry differences. In single-regression analysis, the REM and FEM were used to examine the relationship between individual IVs and the DV without adjusting for the other IVs. For the multivariate regression analysis, to avoid multicollinearity among the IVs and the overfitting problem, the backward elimination method was used to reduce the set of IVs. The Akaike information criterion (AIC) was used to measure the goodness of fit of the models with different sets of IVs (Yum, 2022), with lower AIC values indicating better goodness of fit. As the sample size was relatively small, a corrected AIC (AICc) was used in the analysis. The preceding analysis used complete data to select predictors related to nurses' remuneration. This analysis did not consider AMI in-hospital mortality or hypertension admission rates because of missing data for some countries or periods. However, using the incomplete data, we added these two variables to the models developed in the previous step and examined the relationships between the two quality-of-care indicators and NS level, respectively.

The 28 OECD countries included in this study consisted of two upper-middle-income countries (Mexico and Turkey) and 26 high-income countries, as defined using the economic classification method of the World Bank. We further conducted a sensitivity analysis by restricting the data to the 26 high-income countries to select variables and build multivariable regressions to compare the results with those of the 28 OECD countries.

Stata 14.0 (StataCorp LLC, College Station, TX, USA) was used to develop the REM and FEM. The AICc values for all models were generated using the “MuMIn” package (Version 1.46.0) in R (Version 4.1.2), available under the GNU General Public License V2.0 from the Comprehensive R Archive Network at https://cran.r-project.org/web/packages/MuMIn/index.html.

### Ethical Considerations

The current study used publicly available databases only. The aggregated nature of these databases and the anonymization of all subjects mean that human subjects were not directly involved. Analysis of deidentified public data is not considered human subject research and thus does not require ethical approval by an institutional review board.

## Results

**Figure 1.**
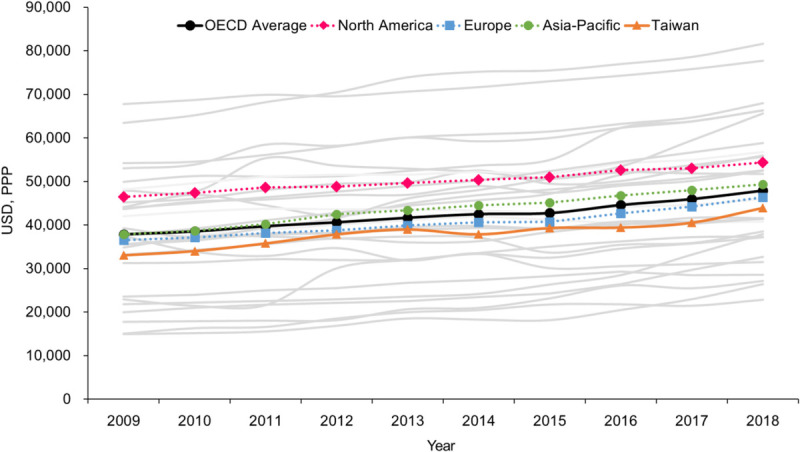
Annual Nurse Salary in 28 OECD Member Countries and Taiwan, 2009–2018 *Note.* OECD = Organisation for Economic Co-operation and Development; USD, PPP = U.S. dollars (USD) corrected for purchasing power parity (PPP). The gray lines represent the annual salary of nurses for each of the 28 OECD member countries from 2009 to 2018. The filled black circles indicate the average nurse salary of the 28 OECD countries. The dotted lines marked by pink diamonds, green circles, and blue squares represent the average nurse salary of North American countries, Asia-Pacific countries, and European countries, respectively. The orange triangle indicates the salary of nurses in Taiwan.

Annual NS trends for the OECD countries and Taiwan for the period of 2009–2018 are shown in Figure 1. The annual NS level in the OECD countries rose from an average of 37,872 USD (PPP) to 47,886 USD (PPP) over this period, representing an increase of 26%. This level in Taiwan rose over the same period from an average of 33,030 USD (PPP) to 43,920 USD (PPP), representing an increase of 33%. By region, North America had the highest average NS level, followed by the Asia-Pacific region, with Europe reporting the lowest level. During this period, the average NS level in Taiwan was lower than the average for the Asia-Pacific region.

The results of the simple regression model are shown in Table 2. In both the REM and FEM, NS was found to be positively associated with GDP per capita, percentage of high educational attainment, life expectancy at birth, nurse density, physician density, number of physician consultations, number of computerized tomography scanners per million population, and prevalence of obesity (*p* < .05) and to be negatively associated with crude birth rate, infant mortality, bed density, length of stay, household out-of-pocket expenditure as a percentage of health expenditure, prevalence of smoking, and gender wage gap (*p* < .05). Of the examined variables, only hospital discharges and the Gini coefficient were found to be not associated with NS in either model (*p* > .05).

**Table 2.** Results of the Simple Regression Models on the Annual Salary of Nurses of OECD Countries

Variable	Random-Effects Model			Fixed-Effects Model		
	β	*t*	*p*	β	*t*	*p*
GDP	0.58	17.00	.002	0.57	16.17	<.001
AGE65	1.54	3.50	.036	0.87	1.78	.076
CRBIRTHR	−2.43	−8.32	.007	−2.67	−8.77	<.001
HIEDU	1.04	15.73	.002	1.07	15.30	<.001
SMOKER	−1.26	−11.29	.004	−1.25	−10.94	<.001
ALCHCONS	−1.18	−2.11	.085	−1.62	−2.48	.014
OBESEP	1.98	12.47	.003	2.11	12.84	<.001
NURSENO	1.62	5.92	.014	1.51	4.89	<.001
DOCTORNO	9.59	9.84	.005	10.28	10.17	<.001
HBED	−1.94	−3.60	.035	−2.21	−3.45	<.001
CTS	0.69	10.34	.005	0.79	10.87	<.001
EXPHEAL	1.65	3.41	.038	0.72	1.30	.196
OOPP	−0.88	−5.03	.019	−0.88	−4.23	<.001
CONSULTD	1.15	3.11	.045	1.55	3.90	<.001
HDISCHAR	−0.02	−0.73	.271	−0.01	−0.29	.769
LOS	−1.30	−3.01	.048	−1.47	−3.18	.002
CRDTHR	2.04	2.70	.057	3.47	4.01	<.001
INFMORTR	−5.93	−9.84	.005	−6.46	−10.16	<.001
LE	4.02	14.19	.003	4.09	13.72	<.001
GINI_PC	−0.35	−1.48	.139	−0.43	−1.62	.106
GWAGEGAP	−0.47	−3.33	.040	−0.53	−3.50	<.001

*Note.* Number of countries = 28, total number of observations = 280. β = coefficients of regressions; OECD = Organisation for Economic Co-operation and Development; GDP = gross domestic product per capita (expressed in 1,000 U.S. dollars, adjusted to the purchasing power parity, [PPP]); AGE65 = the proportion of the population aged 65 years and over (%); CRBIRTHR = crude birth rate (per 1,000 population); HIEDU = the proportion of the population aged 15–64 years who had tertiary education (%); SMOKER = the prevalence of smoking (%); ALCHCONS = alcohol consumption per capita (liters); OBESEP = the prevalence of obesity, self-reported (%); NURSENO = nurse density (per 1,000 population); DOCTORNO = physician density (per 1,000 population); HBED = hospital bed density (per 1,000 population); CTS = the number of computerized tomography scanners per million population; EXPHEAL = health expenditure as a percentage of GDP (%); OOPP = household out-of-pocket expenditure as a percentage of health expenditure (%); CONSULTD = the number of physician consultations per person; HDISCHAR = the number of hospital discharges (per 1,000 population); LOS = average length of stay in hospitals (days); CRDTHR = crude death rate (per 1,000 population); INFMORTR = infant mortality rate (per 1,000 live births); LE = life expectancy at birth (years); GINI_PC = Gini coefficient (%); GWAGEGAP = gender wage gap (expressed in 1,000 U.S. dollars, adjusted to PPP).

The results of the multivariable regression with backward elimination are shown in Table 3. In the REM, higher NS in the OECD countries was shown to be positively associated with GDP per capita (0.49, 95% confidence interval [CI] [0.41, 0.56]), proportion of population aged 65 years and over (2.72, 95% CI [2.17, 3.26]), crude birth rate (1.02, 95% CI [0.56, 1.49]), number of computerized tomography scanners per million population (0.26, 95% CI [0.17, 0.35]), alcohol consumption per person per year (0.94, 95% CI [0.26, 1.61]), and prevalence of obesity (0.64, 95% CI [0.40, 0.89]) and to be inversely associated with infant mortality (−3.13, 95% CI [−3.94, −2.32]), bed density (−0.99, 95% CI [−1.72, −0.25]), hospital discharges (−0.08, 95% CI [−0.11, −0.05]), household out-of-pocket expenditure as a percentage of health expenditure (−0.34, 95% CI [−0.56, −0.11]), and the Gini coefficient (−0.25, 95% CI [−0.50, −0.01]). Although nurse density was included in the REM to enhance the model's goodness of fit (as determined by the AICc), it was only weakly associated with NS (*p* = .054). For the FEM, the selected IVs' effects on NS were in the same direction and of a similar magnitude as those in the REM.

**Table 3.** Results of the Multivariable Regression Models on the Annual Salary of Nurses of OECD Countries Using the Backward Elimination Method

Variable	Random-Effects Model		Fixed-Effects Model	
	β	[95% CI]	β	[95% CI]
GDP	0.49***	[0.41, 0.56]	0.47***	[0.39, 0.55]
AGE65	2.72***	[2.17, 3.26]	2.76***	[2.17, 3.35]
CRBIRTHR	1.02***	[0.56, 1.49]	0.97***	[0.46, 1.47]
ALCHCONS	0.94**	[0.26, 1.61]	1.40**	[0.69, 2.11]
OBESEP	0.64***	[0.40, 0.89]	0.59***	[0.33, 0.86]
NURSENO	−0.32	[−0.67, 0.03]	−0.16	[−0.52, 0.21]
HBED	−0.99**	[−1.72, −0.25]	−0.68**	[−1.49, 0.14]
CTS	0.26***	[0.17, 0.35]	0.30***	[0.20, 0.41]
OOPP	−0.34**	[−0.56, −0.11]	−0.41**	[−0.66, −0.17]
HDISCHAR	−0.08***	[−0.11, −0.05]	−0.10***	[−0.14, −0.06]
INFMORTR	−3.13***	[−3.94, −2.32]	−3.60***	[−4.45, −2.76]
GINI_PC	−0.25*	[−0.50, −0.01]	−0.34*	[−0.60, −0.07]
Constant	4.03	[−12.58, 20.65]	6.44	[−10.99, 23.86]
ρ	0.97		0.98	
*R* ^2^	.63		.55	
*F*	41.70		79.32	
AICc	5338.10		2978.24	

*Note.* Number of countries = 28, total number of observations = 280. β = coefficients of regressions; CI = confidence interval; OECD = Organisation for Economic Co-operation and Development; GDP = gross domestic product per capita (expressed in 1,000 U.S. dollars adjusted to the purchasing power parity); AGE65 = the proportion of the population aged 65 years and over (%); CRBIRTHR = crude birth rate (per 1,000 population); ALCHCONS = alcohol consumption per capita (liters); OBESEP = the prevalence of obesity, self-reported (%); NURSENO = nurse density (per 1,000 population); HBED = hospital bed density (per 1,000 population); CTS = the number of computerized tomography scanners per million people; OOPP = household out-of-pocket expenditure as a percentage of health expenditure (%); HDISCHAR = the number of hospital discharges (per 1,000 population); INFMORTR = infant mortality rate (per 1,000 live births); GINI_PC = Gini coefficient (%); ρ = intraclass correlation coefficient; AICc = corrected Akaike information criterion.

*p < .05. **p < .01. ***p < .001.

On the basis of the models presented in Table 3, we added two indicators of quality of care to examine their associations with NS using incomplete data. We found an increase in NS to be associated with a decrease in the 30-day mortality rate after admission for AMI (REM: −0.60, 95% CI [−0.93, −0.27]; FEM: −0.69, 95% CI [−1.04, −0.34]). A negative but not significant (*p* > .05) relationship between NS and hypertension hospitalization was also observed (REM: −0.02, 95% CI [−0.03, 0.00]; FEM: −0.01, 95% CI [−0.03, 0.01]).

**Figure 2.**
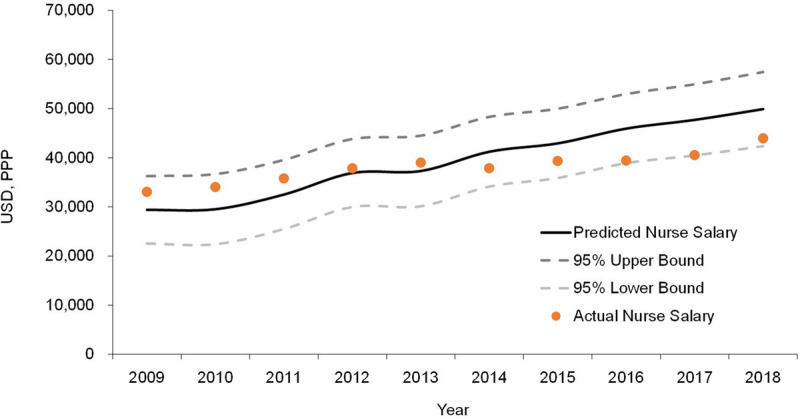
Predicted and Actual Annual Nurse Salary in Taiwan, 2009–2018 *Note.* USD, PPP = U.S. dollars (USD) corrected for purchasing power parity (PPP). The predicted values were drawn from the random-effects model (REM) in the multivariate regression analysis, as shown in Table 3. The solid black line denotes the predicted annual salary for nurses in Taiwan; the dashed lines represent the upper and lower bounds of the 95% confidence interval of the predicted values based on the REM, respectively.

The Taiwan data for each year were introduced into the REM to predict annual NS for the period of 2009–2018. In this prediction, the annual NS for Taiwan increased from 29,390 USD (95% CI [22,532, 36,247]) in 2009 to 49,891 (95% CI [42,344, 57,438]) in 2018. Comparing this prediction with the actual data for Taiwan showed that Taiwan's actual NS for 2018 was approximately 12.0% lower than the model-predicted value (see Figure 2). The sensitivity analysis results for the data from the subset of 26 high-income countries showed the same set of selected variables. The effects of the predictors of the multivariable models remained consistent. The REM based on high-income countries found the actual NS for Taiwan in 2018 to be 10.5% lower than the predicted value.

## Discussion

The results of the analysis on data from the 28 OECD countries for the period of 2009–2018 indicate the following multidimensional factors to be associated with NS: demographics, socioeconomic context, health behaviors and risks, healthcare resources, health financing, healthcare utilization, health outcomes, and economic inequality. To our knowledge, this is the first study to examine country-level correlates of NS using cross-country comparisons.

For every dollar increase in GDP per capita, the average annual NS level was found to increase by an average of $0.46, approximately in line with the labor share of GDP among OECD countries (International Labour Organization & OECD, 2015). GDP is a measure of economic productivity that reflects the value of goods and services created from factor inputs such as labor, whereas GDP per capita is an indicator of the demand for healthcare and the demand for health professionals (Scheffler & Arnold, 2019). Growth in national per capita income increases demand for nurse staffing and thus has a positive effect on nurse wages.

Several demographic factors associated with NS were identified in this study. In general, very young and very old people have the highest healthcare needs (Xu et al., 2007). According to the results of FEM (shown in Table 3), a 1% increase in the proportion of the population aged 65 years and over increases NS by 2,720 USD. On the other hand, a one per 1,000 increase in the crude birth rate is associated with a 1,000-USD increase in NS. The positive associations between these two indicators and NS relate directly to the greater healthcare needs of the very young and old people.

Alcohol drinking, obesity, and smoking are considerable health risks and associated with the prevalence of many chronic diseases, thereby contributing significantly to the disease burden. Countries with a higher prevalence of these lifestyle risk factors were found to have higher rates of noncommunicable diseases and, thus, higher demands for healthcare, which increases demand for nursing care and, consequently, puts upward pressure on nursing wages.

The results of the study revealed the Gini coefficient and the out-of-pocket share of health expenditures to be inversely associated with NS. Social insurance and tax-based welfare schemes are designed to reduce income inequality within a country. As NS in this study includes both social insurance contributions and income taxes, there is an inherent correlation between NS and the Gini coefficient in terms of measuring income inequality. In addition, social health insurance and prepayment financing schemes that reduce income inequality and out-of-pocket payments reduce catastrophic health expenditures, improve families' ability to pay for health services (Xu et al., 2007), and lead to an increase in demand for healthcare.

Nursing represents labor input in the production of healthcare. Raising nursing wages increases the variable cost of healthcare providers' services. As standard economic theory suggests, given a constant demand, an increase in costs on the supply side reduces supply. Thus, nursing earnings and the volume of inpatient care (as indicated by the number of hospital discharges) were found to be negatively related. Meanwhile, the relationship between bed density and NS was profound. On one hand, higher bed numbers require more nursing staff to meet regulations, which positively impacts nursing wages. On the other hand, the number of hospital beds may be regarded as a measure of capital input related to the production of healthcare services. When output is fixed, capital may be viewed as a direct substitute for labor (Reinhardt, 1972). Thus, an increase of capital may be presumed to reduce demand for labor, including nursing staff, which would negatively impact nursing wages. An example of a fixed output is infant mortality, and when this variable was controlled in the multivariable model, the findings indicate that higher bed density is negatively associated with NS.

The use of advanced medical technology increases the demand for nurses. As radiological examinations and interventions become more common, some nursing staff will need to undertake additional radiological training and certification to provide adequate patient care before, during, and after radiological examinations/interventions (Moyo, 2019). Thus, training and certification may be considered investments in human capital that should increase nurse wages.

The relationship between nursing pay and health outcomes is debated. Heyes (2005) suggested that when jobseekers with nursing skills have the opportunity to choose between a nursing job and a job with higher pay but otherwise similar working environments and conditions, those with a “sense of vocation” or motivation to care for people are more likely to select the nursing job. Heyes developed this idea to argue that increasing the pay of nurses would elevate the proportion of those who are financially incentivized to join the profession, which could reduce the quality of care. However, Nelson and Folbre (2006) argued that “sense of vocation” or the motivation to care for people is not a guarantee of high-quality care. Rather, high-quality care requires more education and clinical training, which requires expensive investments in human capital. The results of this study support the theory that higher nurse pay represents a financial reward for providing better quality care and securing better health outcomes, as lower infant mortality was found to be associated with higher NS (after controlling for nurse density).

Likewise, the 30-day mortality rate for patients with AMI after admission was shown to be inversely associated with NS. Patients with AMI need timely and effective care and nursing after admission to the hospital, which can improve the prognosis and chances of survival for these patients (Zhang & Yu, 2021). The in-hospital mortality rate for patients with AMI has been associated with nurse staffing level (Sochalski et al., 2008) and the proportion of registered nurses, who are better educated and more extensively trained than licensed practical nurses (Person et al., 2004). Higher pay for nursing professionals may be expected to help maintain the level of nurse staffing and improve the retention and recruitment of well-trained and skilled nurses. In 2013, Taiwan's 30-day in-hospital mortality rate for patients with AMI was 8.0%, giving the country a ranking of 24th out of 35 OECD countries (including Taiwan; Ministry of Health and Welfare, Taiwan, ROC, 2016). Efforts to improve nurse staffing levels in hospitals and raise the level of nurse training may reduce mortality in patients with AMI and also reflect positively on NSs.

The model based on the OECD country data predicted a 6.1% annual growth rate for NS in Taiwan for the period of 2009–2018 (Figure 2). However, actual NS grew at an annual rate of only 3.2% and was only 1% for the period of 2013–2017. In 2009, the actual salary was slightly higher than the model's predicted value, but by 2016, it had fallen to the lower bound of the model's predicted value. Although the government has financed additional spending on nursing care since 2009, this has not been effective in improving the average salary of nursing staff. These grants were allocated to hospitals rather than nursing staff, and in contrast to the policy's objectives, hospitals may choose not to allocate grant funds to nursing staff (National Health Insurance Committee, 2014). This indicates that more effective policies and strategies to improve NS must be developed by the government.

The COVID-19 pandemic has had a major impact on healthcare systems worldwide. The pandemic raised the demand for healthcare, which in turn increased the nursing workload. Its negative impact on the psychological well-being of nursing professionals further increased nurses' intention to leave the profession (Falatah, 2021). A study using monthly state-level data in the United States found that weekly work hours for nurses decreased by 1% and the weekly wage rate increased by 3% during surges in COVID-19 cases (Butler et al., 2021). Because of data incompleteness, data for 2020 and 2021 were not included in this study. Empirical cross-country comparative research is warranted to assess the pandemic's long-term impact on NS levels and workloads.

There are several limitations to this study. First, data collection approaches and indicator definitions may have differed between the various OECD countries. In addition, the data collection methods and variable definitions used for the Taiwan data were not fully consistent with those used for the OECD data, which reduces the comparability between the model predictions and the actual NS in Taiwan. Second, this study examined cross-sectional associations between the DV and IVs, which cannot be directly interpreted as causal relationships. Third, the omission of several important variables affecting NS such as nurses' average number of years of education and qualifications may have biased the model estimates. Finally, the contribution of total annual hours worked to annual salary was not considered. Therefore, the findings of this study should not be interpreted as indicative of the definite characteristics that influence the wage rates of nurses.

### Conclusions

This study identified, based on data for OECD countries, a number of critical, country-level factors that influence NS. In particular, demand-side factors have a substantial effect on increasing NS. Furthermore, on the basis of model prediction, NS in Taiwan was found to be relatively low in comparison with those in OECD countries. The findings of this study may help policymakers, healthcare managers, and nurse organizations form strategies to improve the remuneration system for nurses in Taiwan.
